# Breast cancer dormancy: need for clinically relevant models to address current gaps in knowledge

**DOI:** 10.1038/s41523-021-00269-x

**Published:** 2021-05-28

**Authors:** Grace G. Bushnell, Abhijeet P. Deshmukh, Petra den Hollander, Ming Luo, Rama Soundararajan, Dongya Jia, Herbert Levine, Sendurai A. Mani, Max S. Wicha

**Affiliations:** 1 Department of Internal Medicine, University of Michigan, Ann Arbor, MI USA; 2 Department of Translational Molecular Pathology, The University of Texas MD Anderson Cancer Center, Houston, TX USA; 3 Center for Theoretical Biological Physics, Rice University, Houston, TX USA; 4 Center for Theoretical Biological Physics and Departments of Physics and Bioengineering, Northeastern University, Boston, MA USA

**Keywords:** Breast cancer, Cancer models, Cancer stem cells, Metastasis, Cancer stem cells

## Abstract

Breast cancer is the most commonly diagnosed cancer in the USA. Although advances in treatment over the past several decades have significantly improved the outlook for this disease, most women who are diagnosed with estrogen receptor positive disease remain at risk of metastatic relapse for the remainder of their life. The cellular source of late relapse in these patients is thought to be disseminated tumor cells that reactivate after a long period of dormancy. The biology of these dormant cells and their natural history over a patient’s lifetime is largely unclear. We posit that research on tumor dormancy has been significantly limited by the lack of clinically relevant models. This review will discuss existing dormancy models, gaps in biological understanding, and propose criteria for future models to enhance their clinical relevance.

## The clinical problem of breast cancer dormancy

**Fig. 1:**
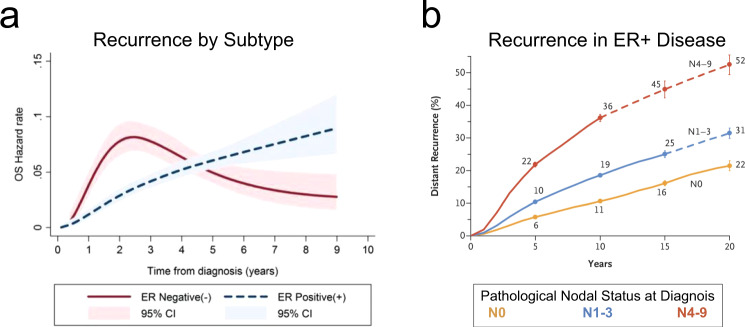
Breast cancer recurrence. Breast cancer recurrence is dependent on (**a**) disease subtype and (**b**) stage at diagnosis. **a** Is reproduced under open access Crown Copyright Oxford University Press from Fig. 2E of Copson et al. 2013^2^. **b** Is reproduced with permission from RightsLink from Fig. 2B of Pan et al. 2017^3^.

Nearly 300,000 new breast cancer cases are diagnosed each year in the USA^1^. Although breast cancer affects many women, those diagnosed with disease localized to the breast have a very favorable prognosis, with a ten-year survival rate above 95%^1^. However, the ten-year survival rate does not fully capture the long-term outcomes for these women. In particular, in women whose breast cancers that express the estrogen receptor (ER^+^), late relapses are common. This contrasts with those with estrogen receptor negative (ER^-^) cancer where the risk of recurrence peaks in year 2 post diagnosis (Fig. 1a). Late relapse in women with ER^+^ breast cancer is thought to result from the activation of dormant tumor cells at metastatic sites such as the bone marrow^2^; and about 20% of women with ER^+^ disease have a recurrence 15 years or more after initial diagnosis^3^. Even more striking is that the relative risk of recurrence is constant over at least 20 years, and this risk is proportional to the initial nodal status (Fig. 1b). These data indicate that women with ER^+^ breast cancer face a lifelong risk of recurrence. The central role of tumor dormancy in mediating these late recurrences highlights the importance of developing effective approaches to target dormant tumor cells. The development of such approaches will depend on a more thorough understanding of the biology of dormancy, including cell-intrinsic and microenvironmental factors that maintain dormancy or that facilitate escape from dormancy. Research focused on tumor dormancy has been limited by the lack of clinically relevant models. This article will review existing in vitro and mouse models of breast cancer dormancy, highlight their advantages and limitations, identify gaps in the current knowledge, and suggest criteria to enhance clinical relevance for future studies.

### Disseminated tumor cells: Clinical samples and systems biology

At present, there is no widely used method to monitor the dormant state (also called minimal residual disease) or to predict the probability of late recurrence in women with newly diagnosed breast cancer. Several multigene expression signatures (Oncotype DX, MammaPrint, Genomic Grade Index) are used clinically to stratify patients based on the risk of recurrence and to inform the use of chemotherapy in early-stage breast cancer^4,5^. Although intrinsic molecular subtypes identified by these assays are predictive of proliferative potential, the risk of early recurrence, and treatment efficacy, they cannot discern the risk of late recurrence. There have been reports that certain molecular signatures of primary tumors can distinguish between early and late recurrence risk, but these have yet to enter clinical practice^6–8^. Furthermore, the minimal overlap between these gene lists, suggests the possibility of overfitting and lack of universal biological relevance^9^.

A more direct assay predictive of tumor recurrence involves the serial monitoring of circulating tumor cells (CTCs) or cell-free DNA in serum samples. CTCs are shed from the primary tumor and/or occult metastatic sites into the bloodstream and can be identified in a simple blood draw. The persistence or appearance of CTCs following adjuvant therapy is associated with significantly lower disease-free and overall survival. The 4-year disease-free survival rate for CTC-negative patients was 92.9%, whereas those with more than 5 CTCs in 30 mL of blood had a 71.9% 4-year disease-free survival^10^. CTCs have also been found in patients who were disease free up to 22 years after mastectomy^11^. This implies that there is a source of tumor cells that are shed into the circulation even when a patient is asymptomatic. Similarly, circulating cell-free DNA with copy number variations associated with the primary tumor have been detected 12 years after diagnosis despite no other evidence of disease^12^. As with CTCs, detection of cell-free DNA is predictive of overall survival and progression-free survival^13^.

Another clinically accessible source of tumor cells in patients with minimal residual disease is the bone marrow. Tumor cells that have disseminated to an organ such as bone marrow are known as disseminated tumor cells (DTCs). DTCs can be detected in bone marrow biopsies or bone marrow aspirates by virtue of their expression of epithelial markers such as EpCAM and pan-cytokeratin. Approximately 60% of ER^+^ patients with metastatic relapse exhibit bone metastasis as the first site of disease recurrence^14^. The propensity of patients with ER^+^ disease to suffer bone metastasis, as well as the presence of DTCs in the bone marrow of breast cancer patients, suggests that DTCs in the bone may be the major source of CTCs and cell-free DNA shedding and may cause late relapse. When one considers the sampling error inherent in taking 1–4 mL of an organ of approximately 3 L total volume^15^ (0.03–0.12%), it is likely that a much higher percentage of patients with ER^+^ breast cancer harbor DTCs in the bone marrow.

Approximately 30% of patients with ductal carcinoma in situ (DCIS) harbor detectable DTCs in the bone marrow^16^, and these patients have a much higher risk of mortality than the general population which persists for at least 15 years following DCIS diagnosis^17^. The number of DTCs and their mutational burden are very similar between patients with ductal carcinoma in situ and with invasive breast cancer^18^. These observations suggest that bone marrow dissemination is an early event in breast carcinogenesis, a finding with important implications for early detection and screening. Although detection of DTCs is associated with an increased risk of recurrence, these cells may remain dormant for long periods: In one study, 30% of patients with detectable DTCs at diagnosis did not suffer cancer recurrence within 15 years^19^.

There are two hypothesized types of cancer dormancy. The cellular dormancy model posits that tumor cells exit the cell cycle and remain in a growth-arrested state. An alternative hypothesis, referred to as tumor mass dormancy, holds that dormancy occurs when the proliferation rate of DTCs is countered by an equivalent rate of cell loss that results in a stable microscopic tumor mass. Support for the first model is provided by several studies that have demonstrated the quiescent nature of DTCs^20^. In contrast, there is little clinical evidence for tumor mass dormancy. Defining the underlying characteristics of tumor dormancy in patients is critical to developing pre-clinical models that accurately reflect this biology.

From a systems biology perspective, dormancy is a phenotypic choice. In order to become dormant and survive in the foreign microenvironment of the metastatic niche, cells must have plasticity; they cannot be irreversibly wedded to a given physiological state but instead must undergo dynamic transitions. Such plastic cells, including cancer stem cells (CSCs), exist in primary tumors^21^ and may arise via de-differentiation during the epithelial-mesenchymal transition (EMT)^22^. Recent work, utilizing computational modeling and direct experimentation, has demonstrated a nuanced connection between EMT status and plasticity. Cells that exhibit hybrid epithelial/mesenchymal (E/M) features^23^ have a higher probability of attaining stemness^24–27^, relative to cells exhibiting pronounced mesenchymal features.

p38 kinase and p21 function have been implicated in the cellular dormancy program, p21 is required to maintain relevant classes of stem cells, and mathematical models of the cell cycle predict that inhibitors such as p21 can block proliferation^28,29^. There is also significant literature on the analysis of circuits that control stemness versus differentiation^30^. Various investigators have formulated network models of drug resistance, for example, to PI3K inhibitors in breast cancer^31^. Finally, there have been major advances in the computational modeling of EMT and how it couples to factors that control stemness^24,32^. Somewhat surprisingly, there has been little effort to put these pieces together to create a quantitative picture of how these circuits interact and how they collectively respond to the chemical and mechanical microenvironment. Thus, to date, computational studies have not adequately addressed the most critical questions facing the dormancy field, such as the key differences between cell autonomous processes and key microenvironmental signals that distinguish between early metastasis or long-term dormancy with the possibility of recurrence. This is a key area for further research that will complement and inform experimental approaches to understand dormancy.

### Dormancy, cancer stem cells, and metabolic plasticity

The functional definition of a CSC is a cell capable of tumor initiation and generation of functional heterogeneous cell populations. There is substantial evidence that breast cancers display a hierarchical cellular organization driven by CSCs^33^. CSCs also drive tumor metastasis and contribute to treatment resistance^33^. Just as normal tissue stem cells are tightly regulated by their microenvironment or “niche”, CSCs that are disseminated in distant tissues may be regulated by signals originating from their niche. This suggests that CSCs entering into or exiting from dormancy may be dependent on the crosstalk between tumor-intrinsic and microenvironmental factors including stromal cells, the vascular system (angiogenic dormancy), and immune cells (immunologic dormancy) as described in the review by Clements et al.^34^.

EMT is a likely contributor to tumor cell dissemination from the primary site, though there has been some debate in the literature as to whether it is absolutely necessary^35^. There is, however, a well-established connection between EMT and the acquisition of stem-like properties^22,24^. Two different subsets of breast CSCs (BCSC) have been identified by our group. Mesenchymal BCSCs (M-BCSC, CD44^+^/CD24^-^) have a slow proliferating, quiescent phenotype, whereas the epithelial BCSCs (E-BCSC, ALDH^+^) are more proliferative^36^. This is consistent with the observation that DTCs in patients have the M-BCSC phenotype indicative of quiescence^37^. We note in passing that whether EMT states are best described as forming a continuum or just a rich spectrum of hybrid versus fully differentiated phenotypes is still uncertain; this does not affect our assertion that as stem-like cells move towards the epithelial end of the spectrum, they adapt their metabolic strategy in well-defined manners. Interestingly, recent evidence suggests these different subsets have different metabolic profiles and differential sensitivity to glycolysis or redox metabolism inhibition^38^. Specifically, M-BCSCs have low levels of reactive oxygen species (ROS) and enhanced glycolytic regulatory enzyme expression, whereas E-BCSCs harbor significantly elevated levels of ROS and enhanced mitochondrial oxidative phosphorylation and deploy NRF2-mediated antioxidant defense mechanisms. Moreover, oxidant stressors (i.e., 2-DG, H_2_O_2_, hypoxia) or antioxidants (i.e., N-acetyl cysteine) are able to induce a transition between M-BCSCs and E-BCSCs. These redox-regulated CSC state transitions are closely linked to the expression or stabilization of key redox-regulated proteins including AMPK, HIF-1, and NRF2.

Computational studies have contributed to our understanding of basic metabolic processes in cancer cells. The most common approach, known as Flux Balance Analysis^39^, aims to solve the fluxes traversing comprehensive networks of metabolic reactions under the assumption that the cell optimizes some predetermined quantity such as biomass production rate. One of the goals of these models has been to explain the Warburg effect, which is the observation that cancer cells are characterized by aerobic glycolysis^40^. These efforts have been only partially satisfactory^41^ as the models need to be augmented by additional somewhat arbitrary assumptions before agreeing with results of experimental efforts^42^. Importantly, computational frameworks are insufficient for addressing some of the most relevant questions that arise in the context of dormant cells. That is, cancer metabolism is adaptive in response to external conditions and changes in cell phenotype. Thus, altering the metabolic state is a crucial part of enabling survival in the dormant stem-like state (as has been readily established in the context of drug resistance^43,44^) and subsequently enabling transitions back to growth and differentiation under specific additional stimuli.

**Fig. 2:**
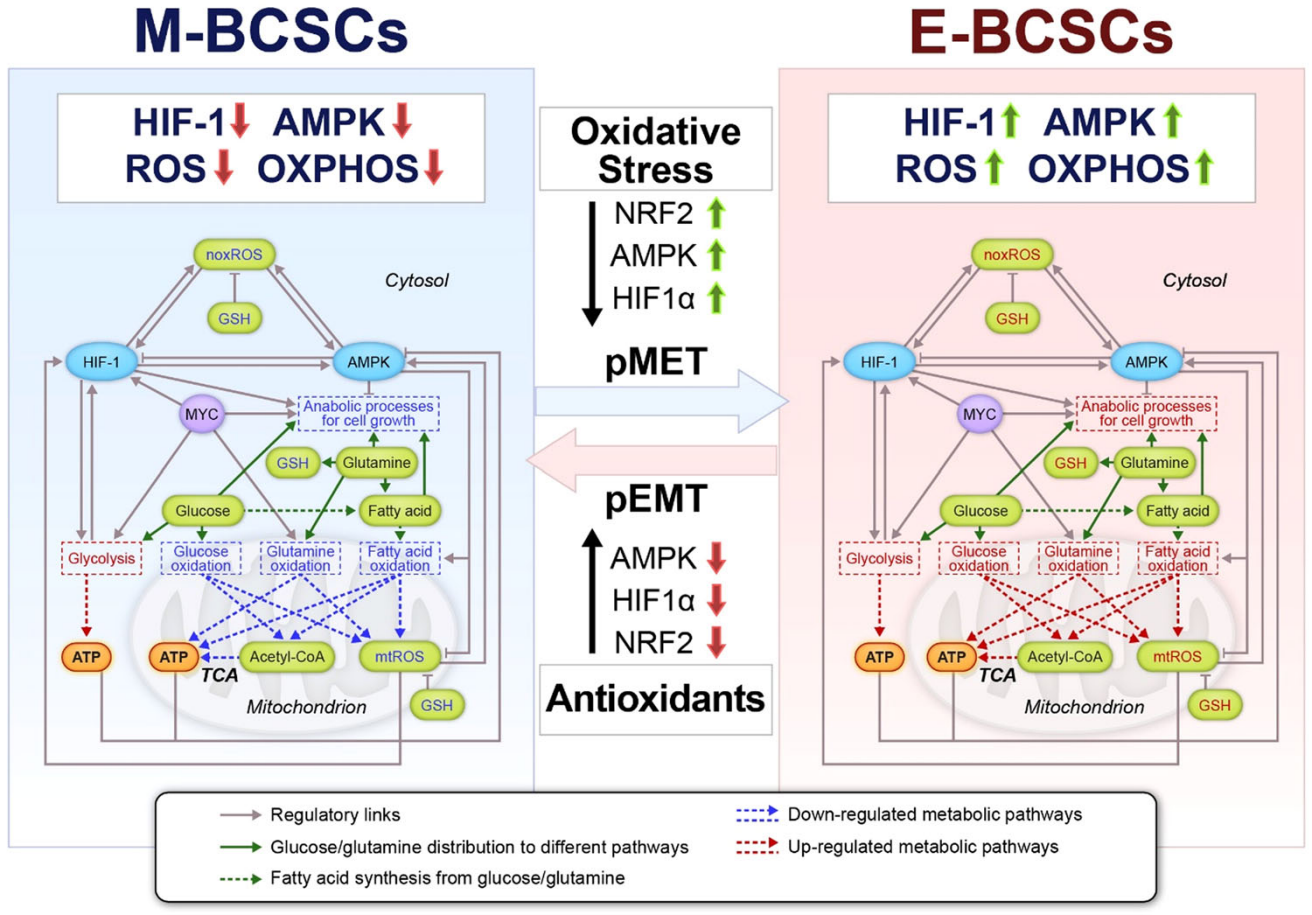
Schematic representation of a metabolic regulatory network simulator. The simulator couples key redox-sensing proteins (HIF-1, AMPK, MYC) with main metabolic pathways (glucose, glutamine, and fatty acid). Gray solid arrows represent positive regulations and gray bar-headed arrows represent negative regulations. Pathways labeled in red are up-regulated and those in blue are down-regulated. The transition from M-BCSC to E-BCSC and the reverse can be induced by alteration of cellular ROS levels. The simulator can be adapted to incorporate additional features to study, for example, the effects of inhibiting antioxidant factors. pMET: partial MET (mesenchymal-epithelial transition, the reverse of EMT); pEMT: partial EMT; mtROS: mitochondrial reactive oxygen species; noxROS: NADPH oxidase-derived reactive oxygen species; GSH: glutathione. This figure is adapted from Jia et al. 2019^45^.

Studying the plasticity of CSCs and DTCs requires the coupling of genetic decision-making circuits to metabolic processes. A mathematical formalism to enable such modeling has recently been proposed with an initial focus on the interplay between the master regulators AMPK and HIF1 in controlling the balance between glycolysis and oxidative phosphorylation (OXPHOS)^45^ (Fig. 2). One of the outcomes of the study was the recognition that cells need not have a binary choice but can mix and match processes to generate needed energy and biomolecular building blocks as they react to local conditions. This model predicted the existence of a metabolically inactive phenotype that is characterized by low levels of HIF-1 and AMPK and low activity of glycolysis and OXPHOS, termed low-low, that was subsequently verified in the context of drug-tolerant melanoma cells^43^. The model also predicts the existence of a hybrid metabolic state in which cells exhibit high levels of HIF-1 and AMPK and high activity of glycolysis and OXPHOS. This hybrid metabolic state has been associated with the hybrid E/M phenotype. Whether the low-low phenotype corresponds to M-BCSCs entry into dormancy in vivo remains to be clarified. Interestingly, the model’s prediction that the antioxidant protein NRF2 is highly expressed in the hybrid E/M phenotype^46^ is consistent with the observation that the levels of NRF2 are high in the proliferative E-BCSCs that effectively uses OXPHOS.

Abundant data have established relationships between EMT and metabolism. For example, the EMT transcription factor SNAIL directly represses expression of the gene encoding the fructose bisphosphatase FBP1^47^. FBP1 repression leads to reduced oxygen consumption and ROS production and to increased glycolysis and biomass synthesis. The fact that EMT can lead to an increased reliance on glycolysis is consistent with findings that partial EMT can lead to stem-like behaviors. The genetic circuits encoding dormancy, stemness, and EMT need to be integrated with metabolism models. Unfortunately, unlike the relative maturity of single-cell transcriptomics, single-cell metabolomics is still in its infancy, and data to build these models will have to be inferred from less direct measurements. It is clear that metabolic switches play important roles in EMT and production of CSCs, that therefore constitutes an area of research crucial for both understanding and targeting dormancy.

### The role of the microenvironment

**Fig. 3:**
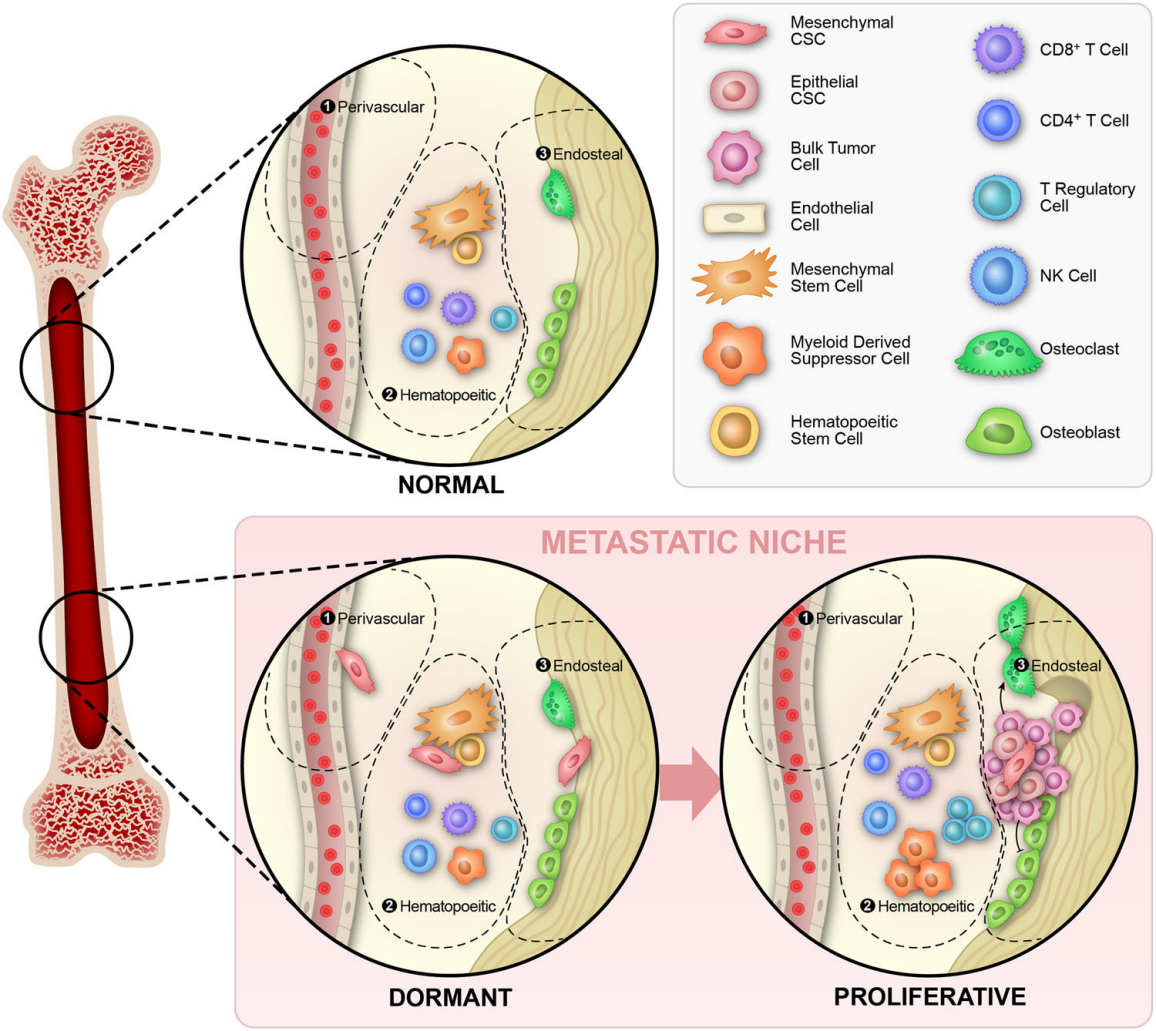
Cellular dormancy in the bone marrow niche. Schematic representation of perivascular, hematopoietic, and endosteal bone marrow niches. It is not well established in humans what niche the tumor cells occupy while in the dormant state. In the proliferative state, bone metastasis is associated with the cycle of bone resorption that occurs in the endosteal niche.

The microenvironment at the metastatic site plays a critical role in the establishment and maintenance of dormancy as well as exit from the dormant state. There is evidence that DTCs occupy various niches in the bone marrow including those generally occupied by developing hematopoietic stem cells^48^, bone endosteal surfaces^49,50^, and perivascular regions^51,52^ (Fig. 3). Evidence for immune regulation of tumor dormancy has been recently reported and summarized in a review authored by Ombrato et al.^53^. It is clear that immune cells provide both stimulatory and inhibitory cues to DTCs, which in turn interact with immune-regulatory cells^54^. From a conceptual perspective, there are two different but connected issues: First, tumor cells are identified as foreign by the effector arms of the immune response^55^ and, second, a complex web of tumor cells of various phenotypes and different immune cells establish the tumor-immune interplay^56^.

Cytotoxic CD8^+^ T cells can recognize cancer cells via MHC-I-dependent presentation of antigens that can arise via mutational events (cancer neoantigens) or over-expression^57^. The recognition of over-expressed factors relies on the lack of perfect negative selection in the thymus^58^. It is likely that both different clones and different phenotypes within a given clone vary in their degree of visibility. For example, it has been established that non-small cell lung cancer cells down-regulate antigen presentation when undergoing EMT^59^. Dormant cells in ER^+^ breast cancer may evade immune recognition, which could be crucial for maintaining a dormant cell population. Various algorithms (the best known is NetMHCpan^60^) based on machine-learning approaches exist that can reasonably predict the binding of peptides to MHC. Also, attempts are underway to devise computational approaches to determine binding specificities between displayed peptides and T cell receptors^61^. To date, none of these new methods have been applied in the context of the differential recognition of dormant versus proliferative cells.

The dynamics of cancer cell recognition by the immune system can be modeled as a population-level competition between the tumor and the immune system. This process has typically been studied using coupled ordinary differential equations (ODEs) that govern the temporal evolution between the populations^62–64^. These models predict a transition between an equilibrium state, where tumor cell proliferation is balanced by immune cell killing (analogous to bulk dormancy), and immune escape where immunity fails. Effects such as T cell exhaustion and therapeutic attempts to counter it can be studied within this context. But there are many limitations. These models typically cannot consider complete cancer cell extinction, requiring attention to effects absent in the ODE treatment. Extensions of the basic model include mixed populations of growing and non-growing cells^65^, but these models neglect the possibility that dormant cells can transition back to a proliferative state. These models also do not consider any spatial aspects of the problem and hence are only applicable to cases where T cell infiltration is not limiting^66,67^. Finally, these models are also highly phenomenological and do not attempt to connect the proposed interaction terms with specific molecular pathways.

When one takes into account the mutual interactions of components of the immune system (the full immune “ecology”), it becomes clear that there can be a large-scale switch in operating mode from “attack” to “recover”, which, in the context of cancer, corresponds to a switch from “anti-tumor” to “pro-tumor” mode. For example, the well-known polarization transition of macrophages from M1 to M2 plays a key role in this functionality switch^68^. Some modeling efforts have attempted to consider this extra level of complexity^56,69^, but these have not been coupled to the phenotypic degrees of freedom relevant in the dormancy problem. Given experimental evidence regarding the role of regulatory T cells as well as myeloid-derived suppressor cells (MDSCs) in setting the immune balance in the metastatic bone niche, this is an important direction for future work. These efforts will be complicated by the influence of systemic factors on the immune interaction with cancer cells. For example, modifications to the immune cells of the bone marrow in patients with untreated breast cancer as well as those undergoing chemotherapy or hormone therapy have been described^70,71^. This suggests that metastases may be influenced by distant primary disease. One such mechanism is the induction of MDSCs^72^ by primary breast tumors. Systemic effects associated with psychological and physiological stress^73^ have also been associated with increased risk of breast cancer relapse^74^. Thus, there is a clear need to extend this modeling framework to address these important aspects.

### In vivo models of breast cancer dormancy

When developing models of tumor dormancy in breast cancer, it is important to critically evaluate their clinical relevance. Bone marrow samples obtained from women with ER^+^ breast cancer provide the most clinically relevant source of DTCs. The use of biopsies that maintain tissue architecture rather than aspirates will facilitate study of both DTCs and their regulatory microenvironment. To maximize their clinical relevance, in vitro and mouse models of dormancy should attempt to recapitulate the biology of the DTC in patients. This includes utilizing ER^+^ models, simulation of a bone marrow microenvironment, and incorporation of an intact immune system. As described below, there are no current breast cancer dormancy models that fulfill all of these requirements. Despite much interest in studying tumor dormancy, the lack of clinically relevant models has limited progress in the field.

To gain insight into tumor dormancy a variety of in vivo mouse models, including genetically engineered mouse models, orthotopic tumor models, tumor resection models, as well as experimental metastasis mouse models have been used^75,76^. For instance, experimental metastasis mouse models have revealed the existence of a dormant state in cancer cells delivered to a metastatic organ site in vivo^77,78^. Upon transplantation into virgin female mice, pregnancy-dependent tumors induced by insertional mutations of mouse mammary tumor virus (MMTV) remain dormant for at least 300 days^79^. Tumor cell dormancy has also been described in transgenic mouse models for breast cancer, in which polyoma middle-T antigen or ERBB2 signaling was studied in mammary gland tissue that was devoid of β1 integrin^80^. The MMTV-based, doxycycline-inducible mouse model (MMTV–rTA; TetO–NEU-NT mice) is a valuable model to study dormancy as well. This model permits the loss of NEU (ERBB2) expression in a pre-established NEU-driven mammary tumor, and therefore provides researchers with a unique tool for study of the molecular mechanisms that control tumor dormancy and re-emergence from the dormant state^81^.

#### ER^+^ models of breast cancer dormancy

Until recently, most studies of hormonal regulation of metastasis have utilized human breast cancer cell lines such as MCF7 or T47D that are implanted into immune-deficient mice (Table 1). These systems have generated important information on hormone-dependent processes, but the necessity of utilizing immunodeficient mice means that these models cannot be used to study the immune system’s role in dormancy. Patient-derived xenograft (PDX) models may recapitulate greater intra- and inter-patient heterogeneity than human cell lines^82,83^, however, these also must be studied in an immunocompromised host^34^. Humanized mice are an emerging technology that may provide a route to better study human cancer in the context of a human immune system^84^, but these have yet to be utilized to investigate metastatic dormancy. In order to circumvent this limitation, mouse models involving syngeneic immunocompetent mice have been developed. The vast majority of these syngeneic mouse breast cancer cell lines have been reported to be ER^-^. However, there is evidence that some of these cell lines have a PAM50 subtype that is Luminal A/B. This is the case for the 4T1 line, which is commonly described as a model of triple-negative breast cancer^85^. It is important to underscore that human breast cancers may be classified as ER^+^ when as few as 1% of cells express ER^86^, though most ER^+^ tumors display >90% positivity^87^. Despite the low expression of ER in tumors with 1% positivity, molecular profiling demonstrates that some display a luminal phenotype and are sensitive to hormone therapy^86^. The same criteria have not been routinely applied to murine breast cancers. To our knowledge only one murine cell line, E0771, has been reported in the literature to have sensitivity to hormone therapy (fulvestrant)^88^. The first ER^+^ mouse mammary carcinoma models that spontaneously and consistently metastasize to the bone were reported using the SSM2 and SSM3 cell lines derived from spontaneous tumors formed in *STAT1*-knockout mice. These models represent a significant advance, since there is a latency period of up to 7 weeks before overt metastases are detected^89^. Dormancy has not been explicitly studied using this model, however. There are conflicting reports in the literature regarding hormone status of murine breast cancer cell lines, which may be due to the use of different methods (immunostaining, PCR, or RNA-seq), lack of expected cross-reactivity between antibodies for ER that are primarily used on human samples, or simply the absence of a standardized cutoff for positivity. We believe this is a crucial problem in the use of murine breast cancer cell lines and suggest it be solved by the following methods. First, two or more methods should be used to identify hormone status (e.g. immunostaining and PCR for ER/PR). Second, appropriate positive and negative controls should be used and reported in these assays (e.g. MCF-7 and 231 cells for a human/mouse cross-reactive antibody, or healthy murine mammary gland tissue). Finally, any murine cell line that is found to be ER^+^/PR^+^ should be tested for responsiveness to estrogen/progesterone in vitro and in vivo via appropriate administration of hormones or hormone therapy.

**Table 1:** Summary of murine breast cancer cell lines as models of breast cancer dormancy.

Cell line/genotype	Strain	Syngeneic	ER status	Luminal A/B	Lung metastasis	Lung dormancy	Bone metastasis	Bone dormancy
T47D^120^	Human	No	Positive^120^	Yes^120^	Yes^121^	Yes 2 weeks^107^	Yes^122^	Yes8 weeks^123^
MCF7^124^	Human	No	Positive^124^	Yes^124^	Yes^125^	Yes9 weeks^126^	Yes^127^	Yes 8 weeks^128^
DB-7^129,130^	FVB/N	Yes	Low^130^	N.R.	Yes^130^	N.R.	Yes^131^	N.R.
Met-1^129,130^	FVB/N	Yes	Negative^85^ Low^130^	Yes^85^	Yes^130^	N.R.	Yes^132^	N.R.
MVT1^133^	FVB/N	Yes	Negative^85^	Yes^85^	Yes^134^	N.R.	N.R.	N.R.
6DT1^133^	FVB/N	Yes	Negative^85^	Yes^85^	Yes^135^	N.R.	N.R.	N.R.
M6^136^	FVB/N	Yes	Negative^85^	No^85^	Yes^136^	N.R.	N.R.	N.R.
HRM-1^137^	FVB/N	Yes	Positive^85^	Yes^85^	Yes^85^	N.R.	N.R.	N.R.
TC11^138^	FVB/N	Yes	Positive^138^	N.R.	Yes^138^	N.R.	N.R.	N.R.
MM51^139^	FVB/N	Yes	Positive^139^	N.R.	N.R.	N.R.	N.R.	N.R.
EO771^140^	C57/BL6	Yes	Negative^85^Positive^141^	Yes^85^	Yes^140^	N.R.	Yes^141^	N.R.
4T1^142^	BALB/c	Yes	Negative^85^Positive^143^	Yes^85^	Yes^142^	No	Yes^142^	No
66cl4^142^	BALB/c	Yes	N.R.	N.R.	Yes^142^	N.R.	N.R.	N.R.
67NR^142^	BALB/c	Yes	Positive^144^	N.R.	Yes^91^	Yes4 weeks^91^	N.R.	N.R.
168FARN^142^	BALB/c	Yes	Positive^145^	N.R.	Yes^146^	N.R.	N.R.	N.R.
4T07^142,147^	BALB/c	Yes	N.R.	N.R.	Yes^142^	Yes 4 weeks^142^	Yes^3^	Yes, with chemo, 5 weeks^148^
EMT6^149^	BALB/c	Yes	Positive^85^	Yes^85^	Yes^92^	Yes 6 weeks^92^	N.R.	N.R.
D2.OR^150^	BALB/c	Yes	Positive^107^	N.R.	Yes^94^	Yes^94,107^ 2–10 weeks	Yes^128^	N.R.
D2.A1^150^	BALB/c	Yes	Negative^85^ Low^107^	Yes^85^	Yes^150^	No^91^	N.R.	N.R.
F311^151^	BALB/c	Yes	Positive^85^	Yes^85^	Yes^85^	N.R.	N.R.	N.R.
TSA/E1^152^	BALB/c	Yes	Positive^85^	Yes^85^	Yes^85^	N.R.	N.R.	N.R.
SSM2^89^	129S6/SvEv	Yes	Positive^89^	N.R.	No^89^	N.R.	Yes^89^	N.R.
SSM3^89^	129S6/SvEv	Yes	Positive^89^	N.R.	No^89^	N.R.	Yes^89^	N.R.
R3T^153^	129S3	Yes	Negative^85^	Yes^85^	Yes^85^	N.R.	Yes^153^	No^153^

For some cell lines, conflicting estrogen receptor status has been reported as noted. Unknown status is indicated as not reported (N.R.). Information is current as of December 1, 2020.

#### Sites of breast cancer dormancy

A number of mouse models of dormancy involve the study of DTCs in the lung. Lung metastasis is readily studied due to the ease of delivery of tumor cells to the lung via tail vein injection as compared to the delivery of tumor cells to the bone marrow via intracardiac or intratibial injection. Although the clinical relevance of the lung as a site of tumor dormancy is unclear, dormancy models in the lung have provided important evidence for immune regulation of dormancy. For example, De Lara et al. demonstrated that the CD39^+^PD-1^+^ subtype of CD8^+^ T cells mediated metastatic dormancy in breast cancer using the syngeneic mouse lines 4T07 (dormant) and 4T1 (non-dormant)^90^. Syngeneic mouse models with dormancy phenotypes and manipulation of the immune background in transgenic mice represent a powerful tool to investigate immune regulation of dormancy.

### Mechanisms of breast cancer dormancy in established immunocompetent murine models

Table 1 summarizes the characteristics of the existing possible in vivo models of dormancy and their clinically relevant characteristics. All cell lines included are murine, to facilitate the study of dormancy in a fully immunocompetent organism, with the exception of MCF7 and T47D which are shown to illustrate ER + cell lines that demonstrate bone metastasis and dormancy. Of these murine breast cancer cell lines, 8 of 22 demonstrate metastasis to bone while 19 of 22 demonstrate metastasis to the lung. Of those 19 that metastasize to lung, 4 have demonstrated dormancy in the lung. Interestingly each cell line (67NR, 4T07, EMT6, and D2.OR) has demonstrated some degree of immune dependence for the mechanism of lung dormancy. Specifically, 67NR dormancy can be abrogated by LPS administration^91^, 4T07 dormancy is controlled by CD39 + PD-1 + CD8 + T cells^90^, EMT6 lung dormancy is controlled by the interplay between granulocytic-MDSC and CD8 + T cells^92^, and D2.OR cells can be awakened from dormancy via LPS administration^93^ or induction of fibrosis^94^. Only one study has demonstrated dormancy in the bone marrow in an immunocompetent host using chemotherapy treatment of 4T07 cells and identified the role of the perivascular niche in DTC chemotherapy resistance^52^. These findings highlight the importance of the immune system and use of immunocompetent models in the study of breast cancer dormancy.

### Challenges for in vivo models of tumor dormancy moving forward

Although mouse models have provided valuable information on the regulation of tumor dormancy, all the existing models have limitations. There are currently no in vivo models that faithfully replicate the clinical situation of dormant breast cancer cells residing in the bone marrow of a fully immunocompetent host. The development of models that are ER^+^, immunocompetent, and dormant in bone marrow are of the utmost importance for the field moving forward.

### In vitro models of breast cancer dormancy

**Fig. 4:**
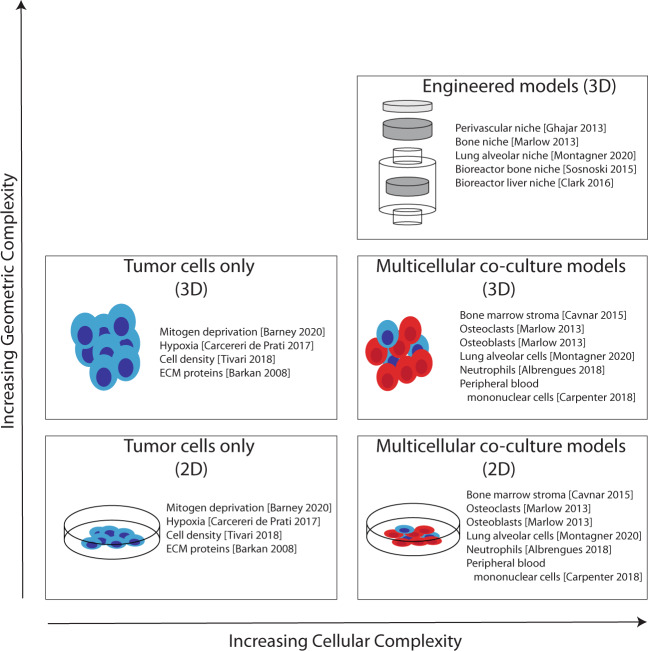
Schematic representation of in vitro models of dormancy that increase complexity of geometric and cellular components. Engineered 3D models generally exhibit the greatest geometric and cellular complexity combining both multicellular models and engineered 3D geometry.

Understanding how DTCs remain dormant and how environmental cues awaken them is essential for developing novel treatment approaches. Study of DTCs has been hampered, in part, by the lack of in vitro experimental models that recapitulate the bidirectional interactions of DTCs and their complex, dynamic microenvironment. Some of the in vitro experimental models widely used to study tumor dormancy and strategies to increase the complexities of these in vitro models to more faithfully mimic clinical dormancy are discussed in this section (Fig. 4).

#### 2D and 3D monoculture models

A simple model of tumor cell dormancy in vitro can be generated using mitogen (i.e., serum) deprivation of 2D cultures for an extended period (up to 14 days), which selects for a quiescent population. With this model, Barney et al. recently demonstrated a role of TGFβ-mediated fibronectin deposition in the promotion of the FAK-ERK survival signaling axis and maintenance of a dormant state^95^. In another study, Tivari et al. developed a 2D in vitro dormancy model of ER^+^ MCF7 cells by plating at clonogenic density on fibronectin-coated surfaces to select for a quiescent population in the presence of FGF-2^96^. In addition to fibronectin and FGF-2, chronic hypoxia or cobalt chloride treatment of metastatic breast cancer cells MDA-MB-231 or ER^+^ MCF7 cells has been reported to induce reversible quiescence or dormancy^97,98^. Although these models are useful for investigating the molecular mechanisms that result in the establishment and survival of dormant cells, 2D culture models often do not recapitulate in vivo findings due to the lack of relevant cell–cell and cell–matrix interactions that only occur in 3D. 3D Cultrex® basement membrane extract has been employed to model in vivo growth characteristics of cells that exhibit either dormant or proliferative metastatic behavior in vivo in more biologically relevant 3D culture^94,99^. Models using transglutaminase crosslinked collagen gels^100^, fibrin hydrogels^101^, tunable PEG hydrogels^102^, and engineered poly(ε-caprolactone) scaffolds with aligned or random fibers^103^ have also been developed to model breast cancer dormancy in 3D culture. The selection of specific subtypes of tumor cells is another method for studying dormancy in vitro. For example, M-BCSCs have been shown to represent a more quiescent subpopulation of tumor cells than E-BCSCs or bulk tumor cells^36^.

### Multicellular co-culture models

To model tumor dormancy in the metastatic bone niche, Marlow and colleagues reported a multicellular co-culture model. Their models are based on co-cultures of breast cancer cell lines in 3D collagen biomatrix with HS-5 human bone marrow stromal cells, which generate a supportive niche, or multicellular stromal cells consisting of HUVEC, fetal osteoblasts, and HS-5, which generate an inhibitory niche^104^. Similarly, by creating organotypic models of lung or bone marrow microvascular niches consisting of lung fibroblasts or mesenchymal stem cells along with HUVEC, Ghajar et al. showed that stable microvasculature constitutes a dormant niche, whereas sprouting neovasculature sparks micro-metastatic outgrowth of breast cancer cells^51^. Although these 3D co-culture systems support quiescent breast cancer cells, the assay format used in these models precludes large-scale screening of compounds that selectively inhibit growth of dormant tumor cells. To overcome this issue, Cavnar et al. reported the development of a 384-well 3D spheroid model in which cancer cells are reversibly arrested in the G1/G0 phase of the cell cycle due to co-culture with bone marrow stromal cells; this system was used to model selective elimination of dormant tumor cells from bone marrow^105^.

#### Tissue-engineered models

The rapid development of sophisticated tissue-engineered ex vivo biomimetic platforms together with co-culture of multiple types of tissue stromal cells and cancer cells have led to a better understanding of how the bone, liver, and lung alveolar microenvironments regulate breast cancer dormancy and reactivation. This emerging area of research is the focus of a thorough literature review from Montagner et al.^106^. For instance, using metastasis-indolent MDA-MB-231BRMS1 cells co-cultured in osteoblast bioreactors that generate a multilayered bone-like structure, Sosnoski et al. showed that cancer cells attached to the bone matrix produced by MC3T3-E1 osteoblasts are dormant until addition of bone-remodeling mediators including TNFα, IL1-β, IL6, and PGE2, which stimulated cell proliferation^50^. Using lung alveolar type 1 and type 2 cells and fibroblasts co-cultured with the metastatic indolent D2.OR mouse mammary tumor cells on a gas-permeable substrate in mitogen-low glucose-low medium, Montagner et al. demonstrated that the indolent behavior of D2.OR cells in the lung is determined by their interaction with alveolar epithelial cells, in particular the type 1 cells. The indolent behavior is mediated by frizzled-related protein SFRP2, which is secreted by alveolar epithelial cells. SFRP2 promotes the formation of fibronectin fibrils by indolent cells to drive integrin-dependent pro-survival signals^107^. Similarly, a 3D hepatic micro-physiological system that reproduces several features of liver physiology results in spontaneous dormancy in a subpopulation of breast cancer cells^108,109^. These ex vivo models of breast cancer dormancy allow investigators to mimic the pathophysiology of metastatic breast cancer cells more reliably than standard 2D cell culture systems. Notably, due to physiologically relevant cell–cell and cell–extracellular matrix interactions that occur in the ex vivo systems, cells in these models exhibit several different phenotypes of tumors generally not observed in cells cultured in 2D or 3D.

#### Challenges for in vitro models of tumor dormancy

Dormant breast cancer cells usually reside in specific microenvironments such as the bone endosteal surfaces^49,50^ and perivascular regions in the bone marrow, lung, and brain that are infiltrated by immune cells^51,52^. However, despite the progress made in the development of complex, multicellular, or tissue-engineered systems to model tumor dormancy, none of these models have incorporated immune cells. Given the importance of immune surveillance in regulating tumor behaviors, future studies will need to incorporate immune cells into organotypic or organoid cultures^110^. Such co-culture systems have been recently developed^111^, although not yet utilized to study tumor dormancy. The integration of immune cells into organoid models will be of great interest and utility for the study of dormancy in both the primary tumor microenvironment and various metastatic microenvironments that may be able to be recapitulated with organoid systems in vitro.

### Breast cancer dormancy models that bridge the gap between in vitro and in vivo systems

Successful integration of in vitro and in vivo systems remains a challenge for tumor dormancy research. Three models have been described that incorporate features of both in vitro and in vivo dormancy models. The first was developed by the Dontu lab and combines human osteoblasts, endothelial cells, and tumor cells in a 3D collagen scaffold as mentioned above^104,112^. Crucially, the 3D scaffold facilitates the implantation of these dormant microenvironments into the subcutaneous space of mice, resulting in development of an inhibitory (i.e., dormant) or a supportive niche in vivo. The second of these models was developed by the Shea lab and utilized a microporous polymer scaffold and implantation into tumor-bearing mice to generate a niche in vivo to which metastatic tumor cells home^113–115^. This approach facilitates the development and study of the metastatic niche in vivo and can be expanded via explantation of the engineered niche and subsequent culture in vitro^116,117^. The third model developed by the Lee lab utilizes a combination of the approaches^118^. In this model, pre-seeded human bone marrow stromal cells form a vascularized niche after implantation to which both human peripheral blood mononuclear cells and prostate cancer cells home in vivo. These engineered niches can be explanted and monitored for tumor cell growth or dormancy ex vivo. Taken together, these models bridge the gap between in vivo and in vitro microenvironments and provide unique opportunities to study how systemic cues resulting from primary tumor development, aging, or immune dysfunction promote or inhibit dormancy on a whole organism scale.

### Future directions

**Fig. 5:**
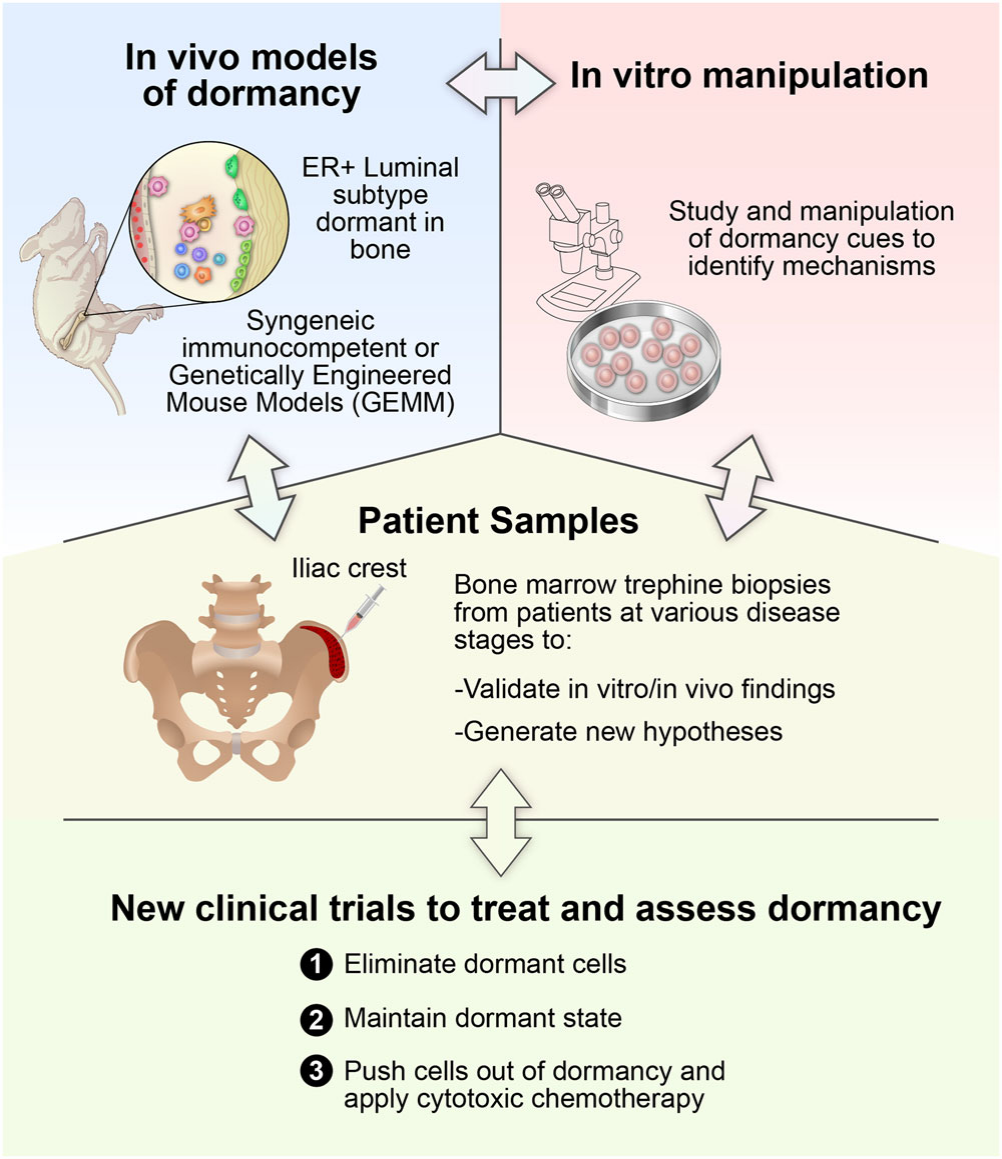
Proposed integration of subfields of breast cancer dormancy research moving forward. The successful study of breast cancer dormancy relies on the interplay between in vivo models of dormancy, in vitro manipulation, and patient samples in order to identify mechanisms of dormancy, generate new hypotheses, and develop therapies with the ultimate goal of new clinical trials to study breast cancer dormancy and to assess treatments.

Tumor dormancy is a major clinical problem particularly relevant to ER + breast cancer. Most current therapies do not kill non-dividing dormant cancer cells. One therapeutic alternative is to force dormant cancer cells to exit dormancy and then target them as they proliferate. However, this approach depends on the ability to kill all cells as they escape dormancy. Failure to accomplish this may increase the likelihood of metastasis. Theoretically, treatments that maintain the dormant state could be developed. This was the rationale for extending the period of adjuvant hormonal therapy from 5 to 10 years^119^. Although this extended therapy may delay recurrence in some patients, it is often associated with side effects. The development of effective strategies to target dormant cancer cells raise additional issues: First, it is not clear how to identify patients who require such treatment after completion of adjuvant therapy as well as how to monitor therapeutic efficacy. Future studies need to evaluate the utility of non-invasive assays such as those that detect CTCs or cell-free DNA for identification of women with dormant cancer cells. Second, DTC assessment requires invasive and painful biopsies but may provide sufficiently worthwhile information to justify use. It remains to be determined whether women with pure ductal carcinoma in situ are more likely to harbor dormant cells than women without cancer cells in the ducts. Finally, methods to assess elimination of DTCs must be developed. The complexity of tumor dormancy and the difficulty of conducting clinical trials over two or more decades highlights the need for continued pre-clinical model development (Fig. 5). As highlighted in this review, current models, although valuable, all have limitations. Future development of more clinically relevant models will help elucidate the biology underlying breast cancer dormancy and develop strategies to overcome the clinical challenge of breast cancer relapse.

## Data Availability

No data or code are associated with this manuscript.
